# ATR kinase inhibitors NVP-BEZ235 and AZD6738 effectively penetrate the brain after systemic administration

**DOI:** 10.1186/s13014-018-1020-3

**Published:** 2018-04-23

**Authors:** Guido Fròsina, Aldo Profumo, Daniela Marubbi, Diana Marcello, Jean Louis Ravetti, Antonio Daga

**Affiliations:** 1 Mutagenesis & Cancer Prevention, Ospedale Policlinico San Martino, Genoa, Italy; 2 Biopolymers and Proteomics, Ospedale Policlinico San Martino, Genoa, Italy; 3 Department of Experimental Medicine (DIMES), University of Genova, Genoa, Italy; 4 Pathological Anatomy and Histology, Ospedale Policlinico San Martino, Genoa, Italy; 5 Regenerative Medicine, Ospedale Policlinico San Martino, Genoa, Italy

**Keywords:** Ataxia Telangiectasia and Rad3 related protein, pharmacokinetics, blood brain barrier

## Abstract

Ataxia Telangiectasia and Rad3 related protein (ATR) is a central mediator of the response to DNA damage that may cause the quiescent resistance of cancer initiating cells to genotoxic radiotherapy. NVP-BEZ235 is a dual PI3K/mTOR inhibitor that also effectively targets ATR with IC_50_ = 21 × 10^− 9^ M in cells. AZD6738 does not target significantly PI3K/mTOR-related kinases but specifically inhibits ATR with IC_50_ = 74 × 10^− 9^ M in cells. Both drugs have been proposed as radiosensitizers of different tumors including glioblastoma (GB), the most malignant brain tumor. In order to study the radiosensitizing properties of ATR inhibitors NVP-BEZ235 and AZD6738 towards GB, we have preliminarily investigated their capacity to penetrate the brain after systemic administration. Tumor-free CD-1 mice were inoculated i.p. with 25 mg/Kg body weight of NVP-BEZ235 or AZD6738. 1, 2, 6 and 8 h later, blood was collected by retro-orbital bleeding after which the mice were euthanized and the brains explanted. Blood and brain samples were then extracted and NVP-BEZ235 and AZD6738 concentrations determined by High Performance Liquid Chromatography/Mass Spectrometry. We found for NVP-BEZ235 and especially for AZD6738, elevated bioavailability and effective brain penetration after intraperitoneal administration**.** Albeit low drug and radiation dosages were used, a trend to toxicity of NVP-BEZ235 followed by ionizing radiation (IR) towards mice bearing primary glioma initiating cells (GIC)-driven orthotopic tumors was yet observed, as compared to AZD6738 + IR and vehicle+IR. Survival was never improved with median values of 99, 86 and 101 days for vehicle+IR, NVP-BEZ235 + IR and AZD6738 + IR-treated mice, respectively. Although the present results indicate favorable pharmacokinetics properties of ATR inhibitors NVP-BEZ235 and AZD6738, they do not lend support to their use as radiosensitizers of GB.

## Background

Glioblastoma (GB) is the most lethal brain tumor with median patients’ survival of 10–14 months [1]. GB recurrence and progression has been linked to specific cell populations [glioma initiating cells (GIC)] refractory to radio-and chemotherapy due to their quiescent state, from which they exit to regenerate the tumor once therapies have ceased [2, 3]. This quiescent state is attributed to constitutive activation of a DNA damage response (DDR), which leads to a number of cellular outcomes including, in a large proportion of tumor cells, cell cycle arrest at the G2/M checkpoint [4]. The constitutively active DDR in GIC may be further elicited by treatment, e.g. RT that, by inducing double strand breaks (DSB) on DNA, typically triggers the DDR. Ataxia Telangiectasia and Rad3 (ATR) related protein is a master regulator of the DDR. Once activated, ATR phosphorylates multiple substrates, including the Chk1 kinase, to regulate cell-cycle progression, replication fork stability and DNA repair. In this context, activation of the G2/M checkpoint acts as a prosurvival mechanism that gives time to the cells to repair their DNA thus reducing the cytotoxicity of RT. Therefore, the ATR pathway has been proposed as a target for developing new drugs that, by inhibiting the DDR, potentiate cytotoxic radiotherapy [5, 6]. As a preliminary step towards determining the radiosensitizing capacity of ATR inhibitors for orthotopic GB induced by primary glioma initiating cells (GIC) in animal models, we have investigated the pharmacokinetics of NVP-BEZ235 (also called Dactolisib or BEZ235), a multiple PI3K, and mTOR and ATR inhibitor and of AZD6738, an orally active and selective ATR inhibitor. We also report the results of a pilot radiosensitization experiment on adult GIC-driven orthotopic GBs .

## Methods and materials

### Chemicals

The ATR inhibitors NVP-BEZ235 (Dactolisib, BEZ235) and AZD6738 were purchased from Selleck Chemicals (Houston, TX, USA, product codes: S1009 and S7693, respectively).

### Pharmacokinetics of ATRi after intraperitoneal (i.p.) delivery

All experiments including animals were performed in compliance with guidelines approved by the Italian Ministry of Health and the committee for animal well-being in cancer research (OPBA) at Ospedale Policlinico S..Martino - Genova, Italy.

Tumor-free 8-weeks old CD-1 mice (Envigo, http://www.envigo.com) were inoculated i.p. with 25 mg/Kg body weight of NVP-BEZ235 or AZD6738. 1, 2, 6 and 8 h later, blood was collected by retro-orbital bleeding after which the mice were euthanized by CO_2_ asphyxiation and the brains explanted. Blood and brain samples were then extracted and NVP-BEZ235 and AZD6738 concentrations determined by High Performance Liquid Chromatography (HPLC)/mass spectrometry (MS) as described [7].

### GIC and orthotopic tumor development

The GIC line COMI has been previously described [7–9]. Briefly, a surgery-derived tumor specimen was obtained, after informed consent, from a 48-year-old male patient with diagnosis of GB, WHO grade IV. The tumor specimen was collected on ice and immediately processed for isolation of GIC according to Svendsen et al [10] Cells were grown in proliferation medium containing EuroMed-N/DMEM/F-12 (Euroclone) and B27 supplement w/o Vitamin A (1:50; Life Technologies), recombinant human FGF-2 (10 ng/mL; Peprotech), and recombinant human EGF (20 ng/mL; Peprotech). Under these conditions, the cells attach and grow as a monolayer in flasks and maintain intact self-renewal capacity for at least 3 months. Removal of growth factors and addition of 10% FCS to the proliferation medium results after 3 weeks in acquisition of astroglial morphology and expression of the differentiation marker glial fibrillary acidic protein (GFAP) [8]. COMI GIC have been characterized in detail by determining their proliferation rate, expression of DDR, stem, *PI3K/Akt* pathway genes as well as the *IDH1, TP53, H3F3A, PDGFRA, CDKN2A and EGFR* status as previously described [7–9]. In particular, these GIC poorly express *TP53* and their *EGFR* locus is amplified as determined by quantitative polymerase chain reaction (qPCR) and Multiplex Ligation-dependent Probe Amplification (MLPA) [7, 9]. Constitutive activation of the DNA damage response with consequent low proliferation rate represent major mechanisms of radio-resistance in COMI GIC, conferring to irradiated cells time for lesion removal or bypass [4, 9, 11]. In order to avoid significant subpopulation selection during prolonged cell culture, COMI GIC samples cultured for no more than two months after post-surgery isolation were used for orthotopic tumor development.

Development and characterization of COMI GIC-driven orthotopic GBs have been previously described [7–9]. Briefly, NOD/SCID mice (4–5 weeks old; Ospedale Policlinico San Martino Animal Facility) were anesthetized with i.m. ketamine and xylazine. Thereafter, the animals were positioned into a stereotaxic frame (David Kopf instruments) and a hole was made using a 21-gauge needle, 2.5 mm lateral and 1 mm anterior from the intersection of the coronal and sagittal sutures (bregma). 0.5 × 10^6^ COMI GIC were injected into the left corpus striatum. Animals were observed daily for neurological symptoms and when moribund were euthanized by CO_2_ asphyxiation. For tumor analysis, animals were euthanized and brains were fixed and stained with hematoxylin/eosin (H/E) or an anti-nestin mouse monoclonal primary antibody followed by a FITC-conjugated goat anti-mouse secondary IgG.

### RT

Whole brain RT of animals bearing orthotopic COMI GB was performed under animal anesthesia obtained by an isoflurane inhalation anesthesia apparatus. Irradiation was performed by an RS 2000 Biological Irradiator (Rad Source Technologies, Alpharetta, GA, USA) equipped with a collimator directing a parallel beam of X-radiation to the head only. The prescription dose was 0.5 Gy. Under those conditions, virtually no radiation to the rest of the body was delivered. The radiation doses were verified by a RadCal Accu-Gold system (Monrovia, CA, USA) equipped with a 10X6–0.6 High Dose Rate Chamber and confirmed by two radiochromic films (Gafchromic® EBT3, Ashland Inc., Covington, KY, USA) placed over and under the mouse body. RT was administered 4 h after each ATRi administration.

### Statistics

Seven mice per treatment group were used. Kaplan-Meier survival curves were compared by both log-rank (Mantel-Cox) and Gehan-Breslow-Wilcoxon tests. The GraphPad Prism 5.01 statistical software was used.

## Results

### Pharmacokinetics

**Fig. 1:**
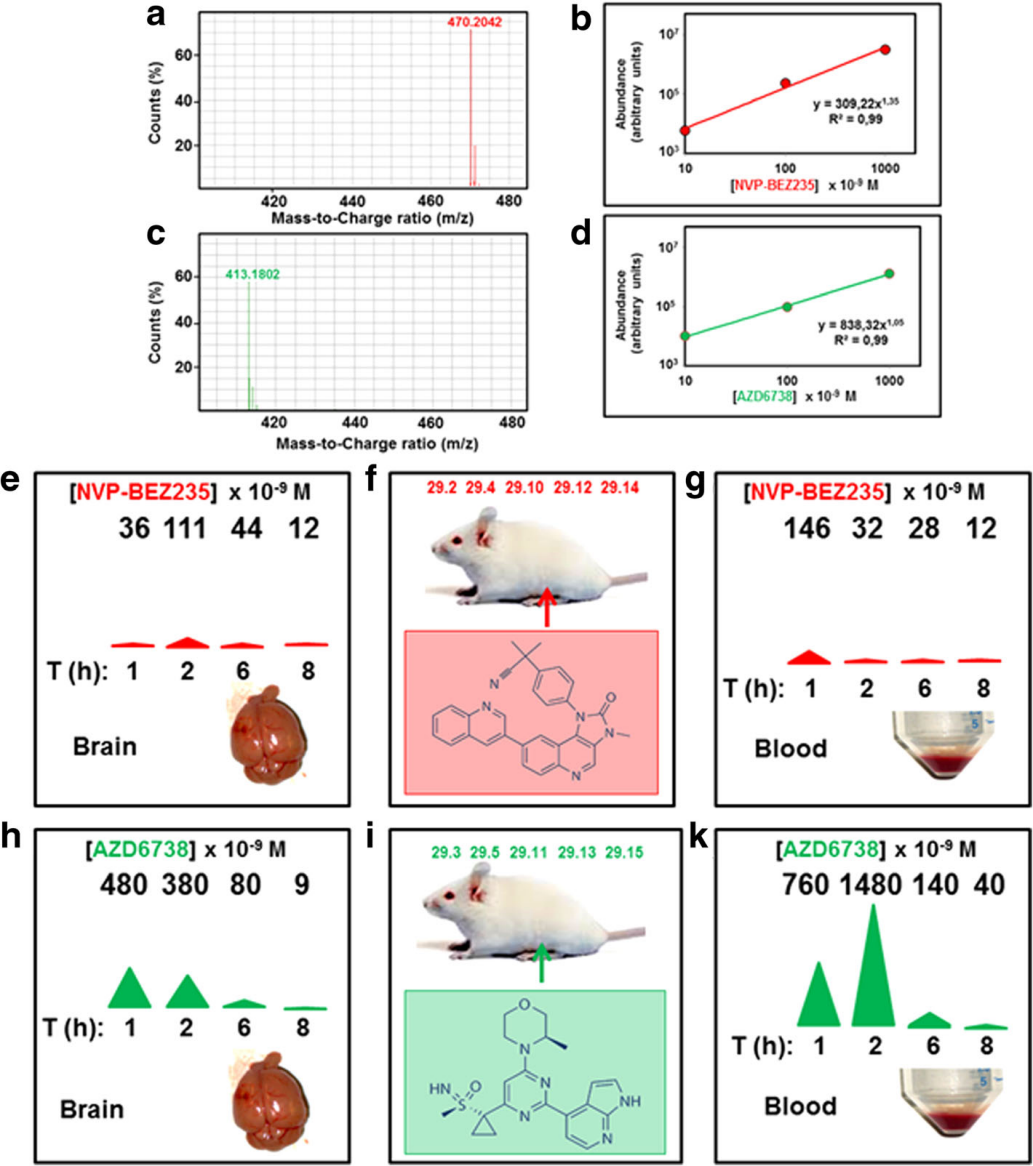
Pharmacokinetics of ATRi studied by HPLC/MS in positive polarity. **a** Isotopic pattern of NVP-BEZ235. **b** Relationship between HPLC/MS-determined abundance and concentration of NVP-BEZ235. The limit of quantitation (LoQ) was 10 × 10^− 9^ M. **c** Isotopic pattern of AZD6738. **d** Relationship between HPLC/MS-determined abundance and concentration of AZD6738. The limit of quantitation (LoQ) was 10 × 10^− 9^ M. **e**-**g** BBB crossing by NVP-BEZ235. Tumor-free mice were inoculated i.p. with 25 mg/Kg body weight of NVP-BEZ235 (**f**). At the indicated times [T (h)] blood was withdrawn retroorbitally for plasma isolation and analysis (**g**). Mice were then euthanized and the brains explanted (**e**). All blood and brain samples were then resuspended in water/methanol, homogenized, centrifuged and the supernatant determined for its NVP-BEZ235 concentration using HPLC/MS as described under Methods and Materials. Concentration values (× 10^− 9^ M) are shown at the top of (**e**) and (**g**) and illustrated at half-panel by the area of a colored triangle. S.D. of values determined by this procedure is on average ± 31%. Mouse ID numbers are shown at the top of F for the sake of reference. **h**-**k** As in E-G but with AZD6738

NVP-BEZ235 inhibits ATR with IC_50_ of 21 × 10^− 9^ M in cells [12]. It also inhibits the PI3K/mTOR pathway with 50% reduction in cells of S473-Akt and T308-Akt levels at concentrations of 8 and 30 × 10^− 9^ M, respectively [13]. AZD6738 is an orally active ATR kinase inhibitor with IC_50_ of 74 × 10^− 9^ M in cells [14]. It does not inhibit significantly related kinases in the PI3K/mTOR pathway [14]. The biodistribution and pharmacokinetics of these ATRi, in particular the concentration reached in the brain after i.p. delivery, is crucial to determine optimal tumor radio-sensitization conditions in vivo. The presence of genuine NVP-BEZ235 and AZD6738 in the mouse blood and brain after i.p. delivery was investigated using HPLC/MS. Figure 1a and 1c show the isotopic patterns of NVP-BEZ235 and AZD6738 respectively, as determined by MS. A logarithmic relationship was found between abundance of NVP-BEZ235 (Fig. 1b) and AZD6738 (Fig. 1d) (expressed in arbitrary units) and their concentrations. For both NVP-BEZ235 and AZD6738, the Limit of Quantitation (LoQ) was 10 × 10^− 9^ M. Tumor-free mice were inoculated i.p. with 25 mg/Kg body weight of NVP-BEZ235 (Fig. 1f) or AZD6738 (Fig. 1i). Mouse ID numbers are indicated at the top of Fig. 1f and I for the sake of reference. Blood samples were then withdrawn retro-orbitally after 1, 2, 6 and 8 h (Fig. 1g and k). Immediately after blood sampling, the animals were euthanized and their brains removed (Fig. 1e and h). All samples were homogenized and extracted concomitantly and its ATRi content determined by HPLC/MS. NVP-BEZ235 reached a blood concentration of 146 × 10^− 9^ M 1 h after i.p. administration and this value decreased > 4-fold (32 × 10^− 9^ M) already at 2 h, indicating rapid excretion of NVP-BEZ235 (Fig. 1g). At this time point, the maximal drug level was observed in the brain (111 × 10^− 9^ M – Fig. 1e), indicating the ability of NVP-BEZ235 to cross the BBB and reach pharmacologically-active concentrations in the brain tissues. This value returned close to the LoQ (12 × 10^− 9^ M) 8 h after the i.p. delivery (Fig. 1e).

AZD6738 showed higher bioavailability as compared to NVP-BEZ235. An AZD6738 concentration of 760 × 10^− 9^ M was measured in the blood 1 h after i.p. administration and this value roughly doubled (1480 × 10^− 9^ M) at 2 h (Fig. 1k). Four hours later (T6) the blood concentration was ten-fold lower (140 × 10^− 9^ M) with further decrease to 40 × 10^− 9^ M at T8, indicating relatively rapid draining of AZD6738 from the blood stream. The highest brain concentration was observed 1 h after i.p. administration (480 × 10^− 9^ M), decreasing to 380, 80 and 9 × 10^− 9^ M after 2, 6 and 8 h, respectively (Fig. 1h). The brain concentrations achievable after i.p. administration of AZD6738 are therefore > 4-fold higher than those achievable with NVP-BEZ235.

### Radiosensitization

**Fig. 2:**
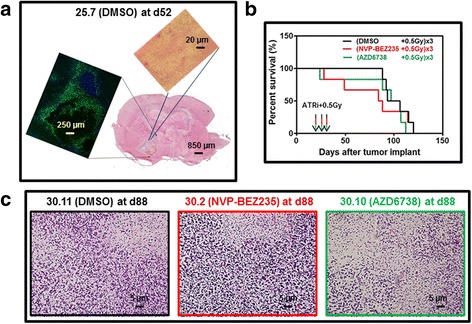
Lack of radiosensitization of orthotopic GIC-driven GB by ATRi. **a** Development of the orthotopic GIC-driven adult COMI GB in NOD SCID mice. Bottom. Coronal section of mouse brain with the orthotopic tumor developed for 52 days. H/E staining. A dimensional bar of 850 μm is shown for reference. Left. Detail of tumor specimen subjected to IHC with antibody specific for the stem cell marker nestin. A large tumor necrosis central region is encompassed by nestin-positive tissue. A dimensional bar of 250 μm is shown for reference. Top. Detail of tumor edge showing infiltration of normal brain parenchyma. A dimensional bar of 20 μm is shown for reference. H/E staining. **b** Kaplan-Meier survival curves of mice treated with ATRì+IR. At d22 of tumor development, *TP53*^−^ COMI tumor-bearing mice were i.p.- injected with 25 mg/Kg body weight of NVP-BEZ235 (red), AZD6738 (green) or vehicle (DMSO-black) followed by irradiation with 0.5 Gy four hours later. This treatment was repeated at d23 and d24. No significant difference in median survival was observed between the three animal groups. A trend towards accelerated dying was observed in NVP-BEZ235-treated animals. **c** Brains of mice treated with ATRi+IR as described under B were explanted and stained with H/E for histopathology analysis at d88. No significant variation of brain histology was observed in mice treated with NVP-BEZ235 + IR (center-red) or AZD6738 + IR (right-green) as compared to DMSO+IR treated mice (left-black)

In 2014, Gil Del Alcazar and coworkers reported that NVP-BEZ235 potently inhibited different kinases of the DNA damage response thus attenuating the repair of IR-induced DNA damage in orthotopic tumors generated by the established GB cell line U87vIII [15]. This resulted in striking tumor radiosensitization, which extended the survival of brain tumor-bearing mice. In 2015, Vendetti and coworkers reported that daily administration of AZD6738 resulting in specific ATR kinase inhibition for 14 consecutive days is tolerated in mice and enhances the therapeutic efficacy of cisplatin in non-small cell lung cancer (NSCLC) xenograft models [14]. AZD6738 can also radiosensitize HCT116 colorectal subcutaneous tumors by abrogating the radiation-induced G2 cell-cycle checkpoint and inhibiting homologous recombination [16]. Here we wished to investigate whether NVP-BEZ235 and AZD6738 could display similar radiosensitization properties towards GIC-driven orthotopic gliomas. Unlike orthotopic tumors developed from established GB cell lines, the COMI primary GIC-driven orthotopic GB faithfully recapitulates the growing properties of the clinical tumor including a relatively slow growth rate which leads animals to death in around 100 days (Fig. 2b), infiltration of the normal brain parenchyma as observed after H/E staining (Fig. 2a bottom and top) and a massive positive component for the stem cell marker nestin, as determined by immunohistochemistry with a specific antibody (Fig. 2a left). Figure 2b shows the Kaplan-Meier survival curves of mice bearing COMI GIC-driven orthotopic tumors treated with ATRi plus RT. 22 days after tumor implant, the mice were treated with one i.p injection of 25 mg/Kg body weight NVP-BEZ235 (red), AZD6738 (green) or vehicle (DMSO, black) followed four hours later by 0.5 Gy IR to the head. This combined (ATRi+IR) treatment was repeated two more times at d23 and d24. No improvement of survival of irradiated animals was observed in the presence of ATRi with median animal survivals of 99, 86 and 101 days after DMSO, NVP-BEZ235 and AZD6738 treatment, respectively. In the case of NVP-BEZ235 + IR, a trend towards reduced mice survival was in fact observed (Fig. 2b), suggesting toxicity of three relatively low-dose NVP-BEZ235 administrations of 25 mg/Kg body weight given 24 h apart, each followed by 0.5 Gy IR. No significant histology variations were observed in brain specimens of irradiated animals pre-treated with DMSO, NVP-BEZ235 and AZD6738, indicating that the possible toxicity of NVP-BEZ235 + IR might be exerted towards organs other than the brain (Fig. 2c).

## Discussion

### NVP-BEZ235

Gil del Alcazar and coworkers have described radiosensitization of orthotopic GB by NVP-BEZ235 using the established glioma cell line U87 [15]. The U87-driven tumors yet poorly mimic the infiltrative and neovascularization growth properties of clinical GB [17]. Further, the U87 cell line was recently reported to represent a mix-up with another cell line [18]. In order to investigate the radiosensitization properties of NVP-BEZ235 towards orthotopic GB driven by primary GIC that more faithfully mimic the clinical tumors growth, [17] we preliminarily investigated the pharmacokinetics of NVP-BEZ235 using HPLC/MS. The relatively low dose of NVP-BEZ235 used in this study (25 mg/Kg i.p.) was chosen due to significant toxicity of higher doses towards our animals (data not shown). Consistently, in two independent animal studies utilizing nude rats and NOD/SCID mice in orthotopic xenograft models of GB, Netland and coworkers have found severe side effects of doses of NVP-BEZ235 higher than 25 mg/ml [19]. The early termination of recent NVP-BEZ235 clinical trials due to elevated toxicity may confirm that safety dosing of NVP-BEZ235 should be thoroughly investigated prior to use [20, 21]. Two hours after the i.p. administration of one single 25 mg/Kg body weight dose, NVP-BEZ235 could be detected in the brain of tumor-free mice at concentrations (111 × 10^− 9^ M) significantly higher than the IC_50_ for ATRi in cells (21 × 10^− 9^ M)[12] (Fig. 1e). Hence, In agreement with previous findings, [15, 22] NVP-BEZ235 can diffuse to brain tissues at pharmacologically active levels after systemic administration, albeit the presence of the efflux ABC-transporters (in particular ABCG2) at the BBB may in part restrict its brain penetration [22]. In a preliminary tumor radiosensitization study, we then developed primary adult COMI tumors in NOD SCID mice, whose immunodeficient phenotype is required for reproducible engraftment in virtually 100% of mice (Fig. 2a) [23]. Unlike orthotopic tumors driven by established glioma cell lines such as U87, GIC-driven orthotopic tumors faithfully recapitulate the infiltrating and stem markers-expressing properties of the clinical tumors (Fig. 2a). In order to explore the radiosensitization properties of NVP-BEZ235, we adopted a low-dose protocol with a 25 mg/Kg NVP-BEZ235 i.p. administration followed four hours later by 0.5 Gy, this combination being repeated for three consecutive days. The low doses of IR (0.5 Gy) employed in the present study four hours after ATRi administration were chosen to limit additional toxic effects to the highly radiosensitive NOD SCID mice. Such low IR doses are actively investigated in both the preclinical and clinical settings for GB histotypes [24–29] and their use may be particularly appropriate under radiosensitizing conditions where the killing effect is amplified. Under the above conditions, no elongation of median survival was observed in mice bearing GIC-driven orthotopic tumors treated with NVP-BEZ235 as compared to mice treated with vehicle. On the contrary, a trend towards reduced median survival (86 versus 99 days, respectively) was observed (Fig. 2b). This is consistent with the results by Netland and coworkers who found no survival benefit or inhibition of tumour growth in orthotopic xenograft models of GB developed in nude rats and NOD/SCID mice [19]. Accordingly, the simultaneous treatment with NVP-BEZ235 and the MAPK inhibitor AZD6244 did not lead to synergistic radiosensitization of tumor cells, questioning a radiosensitizing effect of both inhibitors [30]. Albeit the remarkable differences in the animal models and treatment protocols used may explain our failure to reproduce, even partially, the striking radiosensitization results obtained by Gil Del Alcazar and coworkers, our results support the conclusions by Netland and coworkers that the utility in vivo of NVP-BEZ235 is questionable due to toxicity and lack of efficacy towards GB [19]. The early termination of recent NVP-BEZ235 clinical trials due to elevated toxicity and lack of clinical efficacy further support the conclusions of the preclinical studies [20, 21].

#### AZD6738

We also describe for the first time effective brain penetration of AZD6738 using HPLC/MS. AZD6738 reached a concentration of 480 × 10^− 9^ M (Fig. 1h) in brain tissues one hour after one single i.p. administration of 25 mg/Kg body weight, indicating that the concentration of AZD6738 achieved in the brain of our experimental animals exceeded the IC_50_ for ATR kinase inhibition in cells (74 × 10^− 9^ M) [14]. Albeit AZD6738 at 25 mg/Kg body weight administered per os has been reported to enhance the therapeutic efficacy of cisplatin in xenograft NSCLC models, [14] we could not observe any survival improvement of animals bearing orthotopic adult COMI GB treated for three consecutive days with i.p.-administered 25 mg/Kg AZD6738 followed four hours later by irradiation to the head with 0.5 Gy.

In conclusion, although the present results indicate favorable pharmacokinetics properties of ATR inhibitors NVP-BEZ235 and AZD6738, they do not at this stage lend support to a radiosensitizing effect towards orthotopic GIC-driven GB.

## Abbreviations

ATR: Ataxia Telangiectasia and Rad3 related protein
BBB: Blood brain barrier
DDR: DNA damage response
GB: Glioblastoma
GIC: Glioma initiating cells
H/E: Hematoxylin/eosin
HPLC: High performance liquid chromatography
i.p: Intraperitoneal
IHC: Immunohistochemistry
LoQ: Limit of quantitation
MS: Mass spectrometry
NOD-SCID: Non-obese diabetic-severe combined immunodeficient
OPBA: Committee for animal well-being in cancer research
RT: Radiotherapy
